# Clinical features and outcomes of prolonged mechanical ventilation: a single-center retrospective observational study

**DOI:** 10.1186/s40981-019-0284-4

**Published:** 2019-11-04

**Authors:** Isao Nagata, Tetsuhiro Takei, Junji Hatakeyama, Masafumi Toh, Hiroyuki Yamada, Michiko Fujisawa

**Affiliations:** Intensive Care Unit, Yokohama City Minato Red Cross Hospital, 3-12-1, Shinyamashita, Naka-ku, Yokohama, Kanagawa 231-8682 Japan

**Keywords:** Prolonged mechanical ventilation, Intensive care unit, Long-term acute care, Weaning center

## Abstract

**Background:**

Information on epidemiology of prolonged mechanical ventilation (PMV) patients in the acute care setting in Japan is totally lacking. We aimed to investigate clinical features, impact, and long-term outcomes of PMV patients.

**Methods:**

This was a retrospective observational study conducted in a tertiary care hospital. Adult patients who were admitted to our intensive care unit (ICU) from April 2009 to March 2014 and required mechanical ventilation (MV) for ≥ 2 days were included. PMV was defined as having MV for ≥ 21 consecutive days.

**Results:**

Among 1282 MV patients, 93 (7.3%) required PMV, and median duration of MV was 37.0 days. Compared with the non-PMV patients, PMV patients had longer total ICU and high care unit (HCU) stay (34.0 vs. 7.0 days, *p* < 0.001), longer hospital stay (74.0 vs. 35.0 days, *p* < 0.001), and higher hospital mortality (54.8 vs. 21.4%, *p* < 0.001). In multivariable logistic regression analysis, emergency ICU admission and steroid use during MV were associated with PMV. The Kaplan–Meier curves for MV withdrawal and ICU/HCU discharge were almost identical. Among PMV patients, 52 (55.9%) died, 29 (31.2%) were successfully liberated from MV during hospitalization, and 12 (12.9%) still required MV at discharge.

**Conclusion:**

In this investigation, 7.3% of the patients with MV required PMV. Most PMV patients were liberated from MV during hospitalization, while occupying critical care beds for an extended period. A nationwide survey is required to further elucidate the overall picture of PMV patients and to discuss whether specialized weaning centers to treat PMV patients are required in Japan.

## Introduction

Among patients admitted to the intensive care unit (ICU), 30–60% received mechanical ventilation (MV) [1–4]. Although most patients require MV for a short period, a certain proportion of patients require prolonged mechanical ventilation (PMV), and this number has been gradually increasing [5, 6].

In some countries, patients requiring PMV are transferred from ICUs in acute care hospitals to long-term acute care hospitals (LTACHs) or regional weaning centers (RWCs) in the early stage of PMV. These facilities function as specialized post-ICU weaning centers, and approximately 50% of PMV patients are successfully liberated from MV [7–11].

Conversely, in countries such as Japan lacking weaning centers, patients who require PMV may occupy ICU beds in acute care hospitals for an extended period, thereby affecting the fair allocation of critical care resources [2, 4, 12–14]. Furthermore, these patients place substantial loads on intensivists throughout the long period of the weaning trial. Nevertheless, after a total of 21-day stay in ICU or high care unit (HCU), no payment covers these efforts in hospitals that adapt diagnosis procedure combination (DPC) payment system in Japan. Therefore, the concept of post-ICU weaning centers for PMV patients in Japan has recently become a subject of debate [15, 16].

Although a previous multicenter study reported the investigation for patients with chronic PMV in the non-acute care setting in Japan [17], information on epidemiology of PMV patients including how much fraction of MV patients progress to PMV, how long they occupy ICU and hospital beds, and long-term clinical outcomes in the acute care setting is totally lacking. We aimed to investigate the clinical features, impact, and long-term outcomes of patients requiring PMV in the acute care setting.

## Methods

### Study design and population

This investigation was a single-center retrospective observational study conducted from April 2009 to March 2014. Our hospital is a tertiary care medical center located in the center of Yokohama City, comprising 634 beds including 10 ICU and 8 HCU beds. The ICU is a medical–surgical high-intensity ICU with several intensivists working in the day shift and at least one in the night shift. In entire patient population during the 5-year observation period, mean ICU and hospital stays were 3.5 and 12.7 days, respectively. The hospital policy for MV patients is as follows. Patients are initially treated in ICU or HCU by intensivists, and then they are liberated from MV according to our weaning protocol which was based on the guidelines for weaning and discontinuing ventilatory support published in 2001 [18]. Briefly, all MV patients were screened by intensivists whether to initiate SBT every morning (if SBT was indicated, MV patients were conducted SBT) and were evaluated whether to extubate. If MV liberation is difficult and they become stable or a withholding/withdrawal decision is made, they are transferred to general wards with MV and followed by an intensivist-led respiratory care support team.

Patients aged ≥ 18 years who received MV for ≥ 2 days in our ICU were included in this study. PMV was defined as an application of MV for at least 6 h per day for ≥ 21 consecutive days [19]. Successful liberation was defined as being liberated from MV for at least 48 consecutive hours. We excluded patients with chronic home ventilation and those treated with extracorporeal membrane oxygenation. Also, we did not include patients with chronic progressive disease such as amyotrophic lateral sclerosis that were not expected to wean from MV.

The study protocol was reviewed by the Ethics Committee of Yokohama City Minato Red Cross hospital. Because of the anonymous and retrospective nature of this study, requirement of informed consent was waived.

### Data collection

We collected the information of PMV patients (PMV group) from April 2009 to March 2014. To compare PMV patients with non-PMV patients, the information of non-PMV patients from April 2011 to March 2012 (non-PMV group) were collected as representative values.

We collected the following information from electronic medical records of the PMV and the non-PMV group: age, sex, comorbidities, type of admission to ICU, diagnosis at ICU admission, acute physiology and chronic health evaluation (APACHE) II score on the day of ICU admission, steroid use, neuromuscular blocker use, vasoactive drug use, and continuous renal replacement therapy (CRRT) during mechanical ventilation, spontaneous breathing trail (SBT) within 21 days, tracheostomy during MV, time from MV initiation to tracheostomy, length of ICU and/or HCU stay, length of hospital stay, and outcome at discharge. We also collected the following information of the PMV patients: reintubation, reason for PMV and duration of MV, and post-discharge destination. Furthermore, by sending inquiry letters to destination facilities, patient outcomes at 6 months after discharge were collected for those who still required MV at discharge.

### Statistical analysis

Normally or near normally distributed continuous variables were presented as mean values and standard deviation (SD) and were compared using the *t* test. Non-normally distributed continuous data were presented as medians and interquartile ranges (IQR) and were compared using the Wilcoxon rank sum test. Categorical data were expressed as counts and percentage and were compared using the Fisher’s exact test or chi-square test. A *p* value of < 0.05 was considered statistically significant for all comparisons. Multivariable logistic regression analysis was conducted to investigate the factors related to PMV. The following variables were applied to multivariable logistic regression analysis: age, sex, type of admission to ICU, acute respiratory disease, septic shock, APACHE II, and steroid use and neuromuscular blocker use during MV. Data are presented as odds ratios with 95% confidence intervals. Additionally, we drew Kaplan–Meier curves for survival, MV continuation, and ICU/HCU stay to understand general time course and critical care bed utilization of the PMV patients. All analyses were performed using SAS 9.4 (SAS Institute Inc., NC, USA).

## Results

**Fig. 1:**
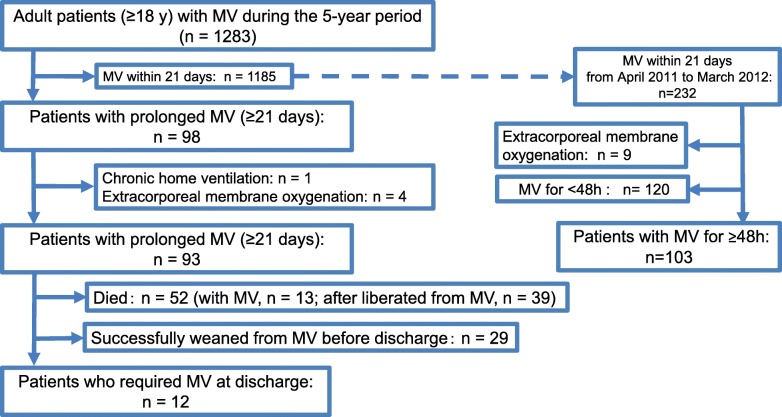
Flow diagram of the study patients. MV, mechanical ventilation

Of the 1283 patients who received MV during the study period, the PMV patients were 93 patients (7.3%). Among 93 PMV patients, 41 (44.1%) patients survived to discharge (29 (31.2%) were successfully liberated from MV and 12 (12.9%) still required MV at discharge) and 52 (55.9%) patients died during hospitalization (39 (41.9%) were liberated from MV and 13 (14.0%) required MV until the time of death). On the other hand, of the 1185 MV patients within 21 days, the non-PMV group was 103 patients (Fig. 1).

Table 1 summarizes the characteristics of the PMV and the non-PMV group. Compared with the non-PMV group, the PMV group had higher rate of emergency ICU admission (95.7 vs. 77.7%, *p* < 0.001) and acute respiratory (46.2 vs. 29.1%, *p* = 0.01) and neuromuscular (4.3 vs. 0.0%, *p* = 0.03) disease in the diagnosis at ICU admission. Also, the PMV group had lower rate of surgical cardiovascular (6.5 vs. 23.3%, *p* < 0.001), medical cardiovascular (6.5 vs. 23.3%, *p* = 0.001), and surgical gastrointestinal (6.5 vs. 16.5%, *p* = 0.03) disease. Age, sex, comorbidities, and APACHE II score were not statistically different between the two groups. In regard to the treatment during MV, the rate of steroid use and tracheostomy was higher (steroid use: 37.6 vs. 18.5%, *p* = 0.003, tracheostomy: 91.4 vs. 5.8%, *p* < 0.001), and the time from MV initiation to tracheostomy was longer (14 vs. 9 days, *p* < 0.02), whereas the rate of SBT within 21 days was lower (40.9 vs. 82.5%, *p* < 0.001) in the PMV group compared with the non-PMV group. PMV group had longer ICU/HCU (34.0 vs. 7.0 days, *p* < 0.001) and hospital stay (74.0 vs. 35.0 days, *p* < 0.001) and higher hospital mortality (54.8 vs. 21.4%, *p* < 0.001) compared with the non-PMV group. Further, in the PMV group, the rate of reintubation within 21 days was 16.1% (15 patients), and median duration of MV was 37.0 days (IQR 27.0–62.0 days). The most common reason for PMV was respiratory disorder (68 patients, 73.1%), followed by central nervous system disorder including cervical cord injury (17 patients, 18.3%), and neuromuscular disorder (7 patients, 7.5%).

**Table 1:** Characteristics of the PMV and the non-PMV patients

	PMV (*n* = 93)	Non-PMV (*n* = 103)	*p* value
Age (years; median [IQR])	73.0 [66.0–79.0]	70 [59.0–79.0]	0.19
Sex (male *n*, %)	64 (68.8)	65 (63.1)	0.40
Comorbidities (*n*, %)			
Heart disease	17 (18.3)	30 (29.1)	0.08
Respiratory disease	9 (9.7)	8 (7.8)	0.63
Renal disease	7 (7.5)	7 (6.8)	0.84
Cerebrovascular disease	12 (12.9)	10 (9.7)	0.48
Type of admission to ICU (*n*, %)			
Scheduled			< 0.001
Elective surgery	4 (4.3)	23 (22.3)	
Emergency			
Ward	33 (35.5)	14 (13.6)	
Emergency department	47 (50.5)	46 (44.7)	
Emergency surgery	9 (9.7)	20 (19.4)	
Diagnosis at ICU admission (*n*, %)			
Surgical			
Neurosurgical disease	0 (0.0)	0 (0.0)	−
Cardiovascular disease	6 (6.5)	24 (23.3)	< 0.001
Gastrointestinal disease	6 (6.5)	17 (16.5)	0.03
Others	1 (1.1)	2 (1.1)	0.62
Medical			
Acute respiratory disease	43 (46.2)	30 (29.1)	0.01
Cardiovascular disease	6 (6.5)	24 (23.3)	0.001
Septic shock	10 (10.8)	5 (4.9)	0.12
Post-cardiac arrest syndrome	10 (10.8)	12 (11.7)	0.84
Trauma	9 (9.7)	5 (4.9)	0.19
Neuromuscular disease	4 (4.3)	0 (0.0)	0.03
Central nervous system disease	2 (2.2)	3 (2.9)	0.78
Others	5 (5.4)	11 (10.7)	0.20
APACHE II score (mean ± standard deviation)	25.6 ± 6.6	24.3 ± 6.6	0.16
Steroid use during MV (*n*, %)	35 (37.6)	19 (18.5)	0.003
Neuromuscular blocker use during MV (*n*, %)	7 (7.5)	13 (12.6)	0.24
Vasoactive drug use during MV (*n*, %)	62 (66.7)	69 (67.0)	0.96
Continuous renal replacement therapy during MV (*n*, %)	13 (14.0)	7 (6.8)	0.10
Spontaneous breathing trail within 21 days (*n*, %)	38 (40.9)	85 (82.5)	< 0.001
Tracheostomy (*n*, %)	85 (91.4)	6 (5.8)	< 0.001
Time from MV initiation to tracheostomy (days; median [IQR])	14 (9–18)	9 (7–19)	0.02
Duration of MV (days; median [IQR])	37.0 [27.0–62.0]	–	–
ICU/HCU stay (days; median [IQR])	34.0 [25.0–49.5]	7.0 [5.0–10.0]	< 0.001
Hospital stay (days; median [IQR])	74.0 [45.0–122.0]	35 [17.0–51.0]	< 0.001
Hospital mortality (*n*, %)	51 (54.8)	22 (21.4)	< 0.001

*PMV* prolonged mechanical ventilation, *MV* mechanical ventilation, *APACHE* acute physiology and chronic health evaluation, *IQR* interquartile range, *ICU* intensive care unit, *HCU* high care unit

The results of multivariable logistic regression analysis for PMV are shown in Table 2. Emergency ICU admission (OR 5.34; 95% CI 1.78–20.0; *p* = 0.006) and steroid use during MV (OR 2.25; 95% CI 1.10–4.70; *p* = 0.03) were the independent factors associated with PMV.

**Table 2:** Multivariable logistic regression analysis for prolonged mechanical ventilation

	Odds ratio (95% CI)	*p* value
Age	1.02 (0.99–1.04)	0.14
Gender		
Male	1.15 (0.60–2.20)	0.67
Type of admission to ICU		
Scheduled	1.00	0.006
Emergency	5.34 (1.78–20.0)	
APACHE II score	1.02 (0.97–1.07)	0.56
Acute respiratory disease	1.00 (0.48–2.06)	0.99
Septic shock	1.58 (0.50–5.61)	0.45
Steroid use during mechanical ventilation	2.25 (1.10–4.70)	0.03
Neuromuscular blocker use during mechanical ventilation	0.54 (0.17–1.66)	0.29

Hosmer-Lemeshow test, 0.94

**Fig. 2:**
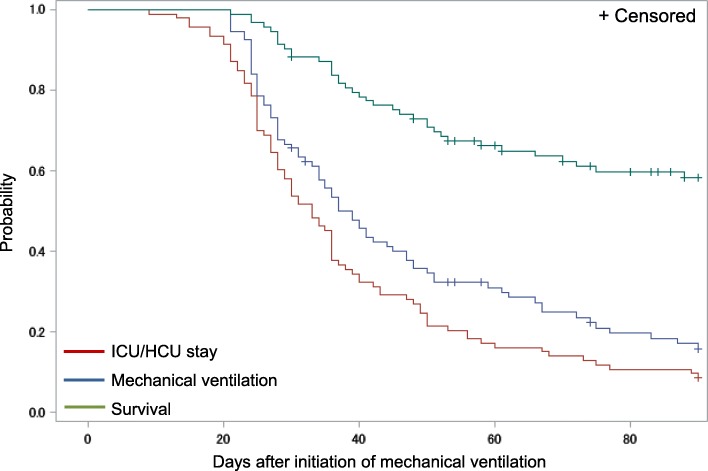
Kaplan–Meier curves for survival, ICU/HCU stay, and continuation of mechanical ventilation (MV) in the patients mechanically ventilated for > 21 days (*N* = 93). The survival curve had a gentle slope from 21 to 90 days after initiation of MV. The curves of MV withdrawal and ICU/HCU discharge were almost identical, having an initial steep slope and transition to a gentle slope in the last third of the curves

Figure 2 shows the Kaplan–Meier survival curve extrapolated by the MV withdrawal and ICU/HCU discharge curves in the PMV group. The survival curve had a gentle downward slope from 21 to 90 days after initiation of MV, whereas the MV withdrawal and ICU/HCU discharge curves initially had a steep downward slope and transition to a gentle downward slope in the last third of the curves, indicating liberation from MV was difficult after approximately 60 days of MV. Furthermore, the MV withdrawal and ICU/HCU discharge curves were almost identical, indicating that the MV patients occupied ICU/HCU beds for a long period until successful liberation from MV.

The patients who still required MV at discharge are listed in Table 3. Their mean age was 73.8 years, and 33.3% of them developed PMV due to cervical cord injury. Patients who developed PMV due to cervical cord injury were seven, and they all had injury in the higher level in cervical cord, which included the fourth cervical cord. Four of them still required MV at discharge. Median duration of MV at our hospital was 90.5 days, and most patients were transferred to long-term chronic care hospital. Mortality rate at 6 months after discharge was 41.6%, and only one patient survived with successful liberation from MV.

**Table 3:** Outcomes at 6 months after discharge of 12 patients who required mechanical ventilation at discharge

Age (years)	Sex	Diagnosis	MV days before discharge	Destination after discharge	Liberation from MV	Six-month outcome
73	M	Acute exacerbation of interstitial pneumonia	107	Chronic care hospital	No	Death
88	F	Ruptured abdominal aortic aneurysm	241	Chronic care hospital	No	Survival
62	M	Post-cardiac arrest syndrome	54	Chronic care hospital	No	Death
80	M	Myxedema coma	114	Chronic care hospital	No	Death
65	M	Cervical cord injury (highest injury site: C3)	118	Chronic care hospital	No	Survival
69	M	Cervical cord injury (highest injury site: C4)	74	Chronic care hospital	No	Survival
74	F	Cervical cord injury (highest injury site: C2)	53	Chronic care hospital	No	Death
83	M	Ruptured thoracic aortic aneurysm	32	Chronic care hospital	Yes	Death
44	M	Pneumonia, HIV	30	University hospital	Yes	Survival
88	F	Cervical cord injury (highest injury site: C4)	58	Chronic care hospital	No	Survival
81	F	Type II respiratory failure	114	Chronic care hospital	No	Survival
78	F	Acute transverse myelitis	237	Home	No	Survival

*MV* mechanical ventilation, *HIV* human immunodeficiency virus

## Discussion

### Brief summary

In this 5-year observational study, 7.3% of the patients with MV required PMV. PMV patients occupied ICU/HCU and hospital beds for an extraordinary amount of time and had higher hospital mortality than non-PMV patients. Emergency ICU admission and steroid use during MV were associated with PMV in multivariable logistic regression analysis. Further, most PMV patients were liberated from MV whether they survived to discharge or not, and 12.9% still required MV at discharge. Six months after discharge, mortality rate of these patients was 41.6%, and only 8.3% survived with successful liberation from MV.

### Relationship with previous studies

According to the previous observational studies, the rates of PMV patients against those with MV were 5.1–28.0% [2–4, 12–14, 20]. In addition, previous studies have shown that PMV patients had a longer duration of MV, ICU stay, and hospital stay compared with those who did not. According to these studies, duration of MV among PMV patients was 29–51 days, ICU stay was 33–52 days, and hospital stay was 51–77 days [2, 4, 12–14, 20]. These studies were conducted in countries lacking the concept of weaning centers, and the results were consistent with those observed in our study.

In our study, emergency ICU admission and steroid use during MV were associated with PMV in multivariable logistic regression analysis. Since few studies have investigated factors related to PMV, we applied the following variables that could potentially affect the development of PMV: age, sex, type of admission to ICU, acute respiratory disease, septic shock, APACHE II score, and steroid use and neuromuscular blocker use during MV, with reference to the factors related to longer duration of MV [21]. In a previous study, the rate of emergency ICU admission in PMV patients was higher than that of non-PMV patients in univariate analysis [14]. Also, previous studies have reported that a large majority of PMV patients were admitted to ICU due to emergency medical reasons including sepsis, respiratory disease, and trauma which is consistent with our study [2–4, 14]. Therefore, emergency ICU admission may be a predictor associated with development of PMV. In addition, although the relationship between ICU-acquired weakness (ICUAW) and steroid use remains controversial [22, 23], steroid use may affect weakness of the respiratory muscle including diaphragm, and thus may prolong the MV duration. However, because the sample size was limited, some confounding factors may exit in the association between PMV and steroid use. Further researches are needed to investigate the risk factor for PMV.

An earlier study in a single RWC showed that the 6-month and 1-year mortality after discharge of patients who still required MV was 65.3% and 78.7%, respectively [11]. A systematic review and meta-analysis reported that pooled 1-year mortality of PMV patients discharged from post-acute care hospitals such as LTACHs was 59% [24]. In our study, 6-month mortality after discharge of PMV patients was 41.6%. Further, it was difficult to compare our study with previous studies [11, 24], due to differences in the research setting. In any case, the long-term mortality of PMV patients was expected to be high. Nevertheless, at present, optimal patient selection at the time of MV initiation remains unclear and is a target for future research.

### Significance and implications

Previous studies conducted in LTACHs and RWCs reported the rate of patients liberated from MV and survived to discharge to be 35.0–54.1% [8–11, 25, 26]. A previous study conducted in an LTACH showed median time to be liberated from MV was 13.5 days, median days on MV before transfer was 29 days, and total duration of MV was 42.5 days [9]. Another multicenter study conducted in LTACHs reported that median time to be liberated from MV was 15 days, median days on MV before transfer was 25 days, and total duration of MV was 40 days [27]. Although research setting was different, these results were similar to those of our study wherein the rate of patients who were liberated from MV and discharged alive against PMV patients was 31.2%; most PMV patients were successfully released from MV within 60 days; and the median duration of MV was 37.0 days. These data indicate that ICUs and HCUs in acute care hospitals in Japan play a role of LTACHs or RWCs.

In our study, MV was applied in ICU/HCU for up to 135 days, which was far longer than the coverage period of ICU/HCU payment (up to 14 days for ICU payment and up to 21 days for ICU/HCU payment in a single admission). Although liberation from MV in PMV patients requires a certain degree of workload on intensivists, most of this effort is not covered by the current payment system in DPC hospitals, consequently burdening acute care hospitals with the economic load. As a result, clinical decisions regarding mechanical ventilation may be made in a less aggressive manner due to financial reasons. Moreover, occupying ICU or HCU beds for an extended period may interfere with admission of other critically ill patients, thereby potentially infringing fair allocation of limited critical care resources [14].

Whether to have specialized centers for PMV patients who need long-term acute care remains contentious. Although PMV patients might be regarded as “outlier” subjects in the ordinary care process of critically ill patients, our results indicated that 7.3% of MV patients were shifted to PMV, which is a substantial proportion. A multicenter investigation performed in the UK reported that establishing weaning centers would potentially reduce acute bed occupancy by 8–10% and overall treatment costs [14]. Because health insurance systems among countries are diverse, this estimation cannot be directly applied to Japan. However, nationwide data demonstrating an overall picture of PMV in Japan is needed.

### Strengths and limitations

To the best of our knowledge, this is the first study to describe the clinical features and long-term outcomes, including post-discharge outcomes, of PMV patients in the acute care setting in Japan. However, our study has several limitations. First, as this study was a single-center study, the sample size was limited, and all the important data that affected PMV may not have been collected. Therefore, it may affect our results of multivariable logistic regression analysis. Second, our hospital is a tertiary care hospital that has an emergency and critical care center and located in the metropolitan area. Our results might be useful to other acute care hospitals with similar characteristics. However, it was not clear if our results apply to hospitals with different features such as academic hospitals comprising only surgical ICUs. Lastly, our ICU comprises a mixed and high-intensity ICU with many board-certified intensivists. Reportedly, not many ICUs in Japan had > 1 intensivists and were managed with high-intensity policy [28]. Therefore, decision-making to initiate MV, managing policy for MV patients, and number of efforts that were applied to PMV patients of other hospitals might differ from those of our hospital.

## Conclusions

Approximately 7.3% of MV patients required PMV, occupying critical care beds for an extended period. A nationwide multicenter study is needed to completely assess the epidemiologic aspects of PMV patients and to determine whether long-term acute care facilities are required in Japan.

## Data Availability

The datasets analyzed during the current study are available from the corresponding author on reasonable request.

## Abbreviations

ICU: Intensive care unit
IQR: Interquartile ranges
LTACHs: Long-term acute care hospitals
MV: Mechanical ventilation
PMV: Prolonged mechanical ventilation
RWCs: Regional weaning centers
